# Biopsychosocial factors during pregnancy and their implications for autism spectrum disorder: a scoping review

**DOI:** 10.1186/s12887-026-06794-7

**Published:** 2026-04-16

**Authors:** Jusselit Estrada, Jasna Ahumada Guzmán, Ana Sol Aguayo, María Angélica Miglino, Mariano del Sol

**Affiliations:** 1 Escuela de Obstetricia, Facultad de Ciencias para el Cuidado de la Salud, Universidad San Sebastián, Concepción, Chile; 2 Doctoral Program in Morphological Sciences, Universidad de La Frontera, Temuco, Chile; 3 Veterinary Medicine. Unimar, Marilia, Brazil; 4 Center of Excellence in Morphological and Surgical Studies (CEMyQ), Faculty of Medicine, Universidad de La Frontera, Temuco, Chile

**Keywords:** Pregnancy, Biopsychosocial, Autism Spectrum Disorder ASD

## Abstract

**Introduction:**

The human brain is part of the central nervous system (CNS) and is responsible for all sensory and motor functions of the body. Regarding its functionality, two types of individuals can be identified: neurotypical individuals, with typical neurological patterns, and neurodiverse individuals, who present variations in their neurological, sensory, communicative, and social characteristics. The last concept includes individuals with autism spectrum disorder (ASD).

**Objective:**

To determine the biopsychosocial risk factors that influence the development of ASD during pregnancy.

**Method:**

A scoping review of articles in the respective research field was conducted in six databases from 2013 to January 2025. A total of twenty-four articles were included, using MeSH/DeCS and open-source keywords. The inclusion criteria were: (i) quantitative, (ii) primary, and (iii) with a confirmed diagnosis of ASD.

**Results:**

Among the biological factors associated with ASD, maternal nutritional status, gestational age and infant birth weight were predominant. During pregnancy and the postpartum period, some of the associated factors were preeclampsia, diabetes mellitus, maternal fever and neonatal asphyxia. Regarding psychosocial factors, stress, depression, and smoking were highlighted, as well as the mother’s years of education. All the aforementioned variables showed an association with the development of ASD in the offspring.

**Conclusions:**

Several biopsychosocial factors are implicated in the development of ASD during pregnancy. This fact highlights its multifactorial cause, as well as the importance of further studying those variables with the greatest implications.

## Introduction

The human brain is characterized by its high complexity and remarkable neuroplasticity. It constitutes the organ that governs all the body’s sensory and motor functions, as well as the means that allows us to interact with the environment and adapt to its changes [1]. In this context, each individual presents particularities that reflect two dimensions or categories. The first category corresponds to the so-called neurotypical individuals, who exhibit neurological patterns considered typical or standard by society. The second category includes neurodiverse individuals, who present variations in brain development and its neurological, sensory, communicative as well as social characteristics [2]. This category includes individuals that present autism spectrum disorder (ASD), also known as autism spectrum condition (ASC). These individuals present a number of persistent difficulties, manifested in the processes of socialization, behavior, interaction and social communication, sometimes including sensory and cognitive variations [3]. The terms ASD and ASC are currently widely used in various countries [4].

It is important to note that the neurological development of the embryo begins around 28 days after fertilization. At birth, the central nervous system is completely formed but not yet fully mature and functional. During the post-natal period, the neurological development is shaped by the life experiences of the individual, which play an important role in fine-tuning the main neural pathways and cortical networks until reaching adulthood [5].

In this regard, the conditions under which pregnancy takes place are critical factors that significantly impact development, including genetic and epigenetic factors that affect the embryo and fetus, as well as the maternal endocrine and immune systems, in addition to a wide variety of possible harmful agents (germs, alcohol, tobacco, drugs, diet, etc.) [6, 7]. As Barker described in his hypothesis on fetal programming [8], it is essential to evaluate the environmental circumstances during pregnancy, as these can significantly influence the embryological and fetal development. These factors can act directly or indirectly, at both the molecular and cellular levels, affecting neural development and brain configuration [8].

Recent studies have shown that various environmental factors during pregnancy, such as chronic illnesses, infections, and even lifestyle choices [9–11] can influence the development of neurodevelopmental disorders.

The causes of autism are not yet fully understood, but they are thought to involve multiple genes and environmental factors, resulting in a wide variety of neurological manifestations.

Global ASD diagnosis figures indicate that the condition occurs in 1 in every 160 births and in Chile in particular, it reaches 1 in every 51 births, which shows a higher prevalence in our country, compared with Colombia, Mexico and the United States [12]. The notable increase in the number of ASC diagnoses underscores more than ever the importance of expanding our knowledge about the multiple causes of autism.

Taking the above into account, the present review explored and evaluated the biopsychosocial risk factors that influence the development of ASD during pregnancy. Understanding these factors could contribute to improved decision-making, education, and public health policies. In the long term, a better understanding of autism will allow for improved early detection, leading to timely medical and psychosocial intervention.

## Material and method

This research was carried out through a Scoping Review type bibliographic review. For this purpose, the following databases were considered: (i) WOS, (ii) Scopus, (iii) SciELO, (iv) PubMed, (v) Science.gov and (vi) LILACS, in a time range that included articles published from 2013 to January 2025. A total of 24 articles were included in the review, using MeSH/DeCS key terms and free terms that addressed the research question: *What are the biopsychosocial factors during gestation that have implications for the development of the autism spectrum condition?*

In the search for key and free terms, the following variables were determined using the mnemonic PIO (Population under study, Intervention, and Outcome, respectively): for the population, *“Pregnancy”* and its synonyms were included; for the intervention, *“Risk Factors”*,* “Epigenomics”* and *“Fetal Development”*, along with their synonyms; and finally, for the outcome, *“Autistic Disorder”* and its synonyms.

The initial search strategy yielded a total of 249 articles, to which the previously defined inclusion criteria were applied. Key terms and their synonyms were joined using the Boolean operator ‘OR’, and the search was subsequently limited using the Boolean operator ‘AND’ to combine the Population, Intervention, and Outcome (PIO) categories.

It is worth noting that the review was included in the international registry of systematic reviews PROSPERO ID CRD420251114066, where details on the search strategy and methodology used can be found.

A total of 16 duplicate articles were identified and eliminated. This allowed us to continue reading the titles and abstracts of a total of 233 articles to determine which met the inclusion and exclusion criteria, which are detailed below:

- Inclusion criteria:
  - ✓ Primary articles
  - ✓ Quantitative articles that determine the statistical relationship and/or risk
  - ✓ Individuals with a confirmed diagnosis of ASD
  - ✓ Social factors
  - ✓ Biological factors
  - ✓ Psychological factors
- Exclusion criteria:
  - ✓ No clear explanation of the statistical analysis of the related variables
  - ✓ Descriptive, qualitative articles

The 24 articles finally selected were evaluated in terms of the study design, methodological quality and interpretation of results, according to the 2015 PRISMA-ScR (Preferred Reporting Items for Systematic Reviews and Meta-Analyses) checklist. PRISMA guidelines were applied to enhance transparency and reporting quality, recognizing that this study corresponds to a scoping review and not a systematic review with meta-analysis. Once the included articles were identified, an Excel data file was created in order to record the analysis of variables for the extraction of data that addressed the research question.

The authors of this review jointly identified the articles and conducted an initial search in the databases previously mentioned. Once the articles were collected, they were reviewed to determine which ones would be included, based on the information provided both in the title and the abstract of the selected articles. This step was conducted independently by two of the authors to avoid potential bias. All of this was performed following the criteria established in the inclusion protocol previously defined by the research team.

**Fig. 1:**
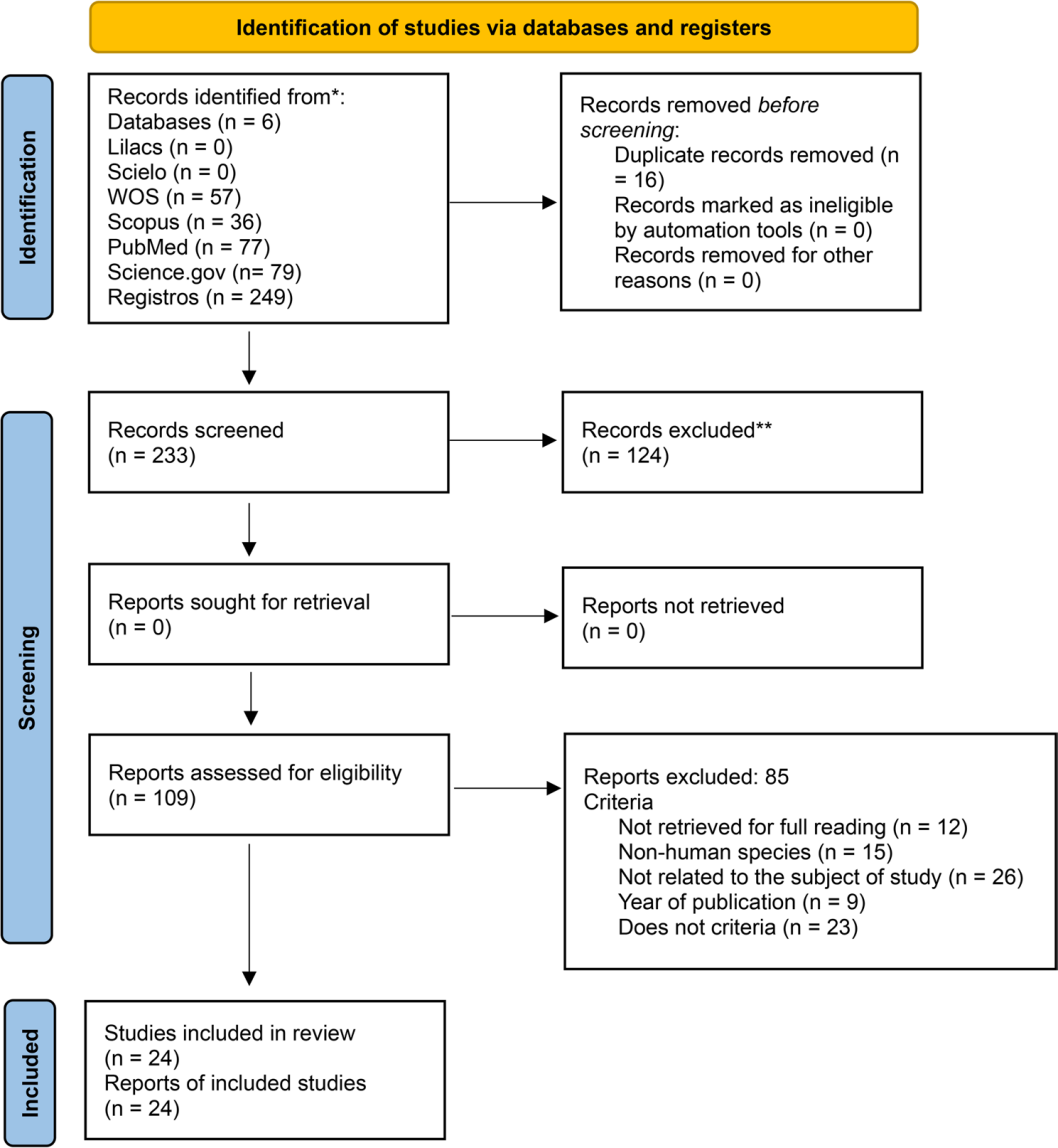
PRISMA flowchart. The stages and all parameters followed in the literature search and selection of the scientific papers used in this study are presented. The number of papers obtained in each substage after applying the inclusion and exclusion criteria is also detailed

The first author, who was not involved in the previous step, was responsible for reviewing those articles in which there were discrepancies between the first two reviewers and those categorized as “possible.” Subsequently, this same author, along with the other two, were responsible for fully reading all articles that met the eligibility criteria. These articles were reviewed in their entirety, and the information thus gathered was used to complete the data extraction matrix. From there, the analysis was conducted, and the results addressing the objectives of the study were written. Figure 1 depicts the details of the stages of the search process in the PRISMA [13] flowchart.

## Results

The results obtained are described below, presented under the categories (A) Psychosocial Factors and (B) Biological Factors. Detailed results are presented in Table 1.

**Table 1:** Prenatal psychosocial and biological factors and their association with autism spectrum disorder/(ASD)

			**STUDY FACTORS**										
			**Psychosocial**				**Biological**						
**Authors**	**Study of type**	**Location**	**Maternal education**	**Substance use**	**Stress and maternal mental health**	**Socioeconomic status**	**Maternal age**	**Nutritional status**	**Gestational age and birth weight**	**Associated pathologies**	**Intrapartum and delivery complications**	**Possible markers**	**Newborn sex**
Fasset M, et al. 2017	Cohort	United States	Higher educational level	Not studied	Mental health pathology	Not associated	20 to 30 years	Not associated	Not associated	Preeclampsia and placental insufficiency.	Dystocic labor due to position	Not studied	Male
Liu X, et al. 2023	Case–control	China	Lower educational level	Multivitamin and folic acid	Not studied	Not studied	Not studied	Not studied	Not studied	Preeclampsia, gestational diabetes	Prematurity	Not studied	Not studied
Brumbaugh JE, et al. 2020	Cohort	United States	Higher educational level	Alcohol and tobacco	Mental health pathology	Not studied	20 to 30 years	Excessive weight gain	Weight and gestational age	Not studied	Prematurity	Not studied	Male
Hisle-Gorman E, et al. 2018	Cohort	United States	Not studied	Topiramate and SSRIs	Mental health pathology	Not studied	Not studied	Obesity	Low birth weight	Hypertension, pregestational infections and GDM.	Complications during childbirth and asphyxia at birth	Not studied	Not studied
Casey S, et al. 2022	Case–control	New Zealand and Ireland	Not studied	Folic acid	Not studied	Not studied	Not associated	Not associated	Not associated	Not studied	Not associated	IL‑17A	Male
Mkhitaryan M, et al. 2024	Case–control	Armenia	Not studied	Magnesium and vitamin B6	Prenatal stress during pregnancy	Not studied	Not studied	Excessive weight gain	Gestational age	Infection and anemia	Not studied	Not studied	Male
Hannon E, et al. 2018	Case–control	Denmark	Not studied	Tobacco	Not studied	Not studied	Not associated	Not studied	Not related to gestational age	Not studied	Not studied	Not studied	Not associated
Brynge M, et al. 2022	Cohort	Sweden	Not associated	Tobacco	Mental health pathology	Second and fourth income quartiles	20–30 years old or over 40 years old	Excess weight	Not studied	Not studied	Not studied	IL-1β, IL-7, IL-13 y MCP-1, GM‑CSF y TNF-α	Male
Courraud J, et al. 2021	Case–control	Denmark	Not studied	Metacholine	Not studied	Not studied	Not studied	Not studied	Not studied	Not studied	Not studied	Metabolomics	Not studied
Ram S, et al. 2019.	Cohort	California	Not studied	Not studied	Lower maternal plasma cortisol levels	Not studied	Not studied	Not studied	Not associated	Not studied	Not studied	Not studied	Male

			**STUDY FACTORS**										
			**Psychosocial**				**Biological**						
**Authors**	**Study of type**	**Location**	**Maternal education**	**Substance use**	**Stress and maternal mental health**	**Socioeconomic status**	**Maternal age**	**Nutritional status**	**Gestational age and birth weight**	**Associated pathologies**	**Intrapartum and delivery complications**	**Possible markers**	**Newborn sex**
Wang LW, et al. 2023	Cohort	Taiwan	Not studied	Not studied	Mental health pathology	Not studied	Advanced maternal age	Pre-pregnancy obesity	Weight and gestational age	SHE-PE, diabetes mellitus, and infection	Antepartum hemorrhage, premature delivery, and cesarean section	Not studied	Male
Talmi Z, et al. 2020	Case–control	Israel	Not studied	Not studied	Not studied	Not studied	Not studied	Not studied	Weight and gestational age	Not studied	Prematurity	Not studied	Male
Iwabuchi T, et al. 2022.	Cohort	Japan	Not studied	Not studied	Not studied	Not studied	Not studied	Not associated	Not studied	Not associated	Not studied	Leptin	Not studied
You J, et al. 2019	Case–control	China	Not associated	Not studied	Not studied	Not studied	Not studied	Not associated	Gestational age	Fetal distress, oligohydramnios, cord loop, ICP, HDP, PE, PP, PROM, HG, GDM	Not associated with mode of delivery	Not studied	No sex difference
Hajj A, et al. 2022	Case–control	Lebanon	Not studied	Not studied	Not studied	Not studied	Not studied	Excessive weight gain	Gestational age	Not studied	Not studied	Not studied	Male
Shieve, et al. 2014	Cohort	United States	Not studied	Not studied	Not studied	Not studied	Not studied	Not studied	Weight and gestational age	Not studied	Prematurity	Not studied	Not studied
Leavey A, et al. 2013	Cohort	Canada	Not studied	Not studied	Not studied	Not studied	Not studied	Not studied	Gestational age	Not studied	Prematurity	Not studied	No sex difference
Persson M, et al. 2020	Cohort	Finland and Norway	Not studied	Not studied	Not studied	Not studied	Not studied	Not studied	Gestational age	Not studied	Prematurity	Not studied	No sex difference
Atladóttir HÓ, et al. 2016	Cohort	Denmark	Not studied	Not studied	Not studied	Not studied	Not studied	Not studied	Gestational age - Extremely premature	Not studied	Prematurity	Not studied	Not studied
Walker CK, et al. 2015	Case–control	United States	Not studied	Not studied	Not studied	Not studied	Not studied	Not studied	Not studied	Preeclampsia and placental insufficiency.	Not studied	Not studied	Not studied

			**STUDY FACTORS**										
			**Psychosocial**				**Biological**						
**Authors**	**Study of type**	**Location**	**Maternal education**	**Substance use**	**Stress and maternal mental health**	**Socioeconomic status**	**Maternal age**	**Nutritional status**	**Gestational age and birth weight**	**Associated pathologies**	**Intrapartum and delivery complications**	**Possible markers**	**Newborn sex**
Patel S, et al. 2021	Cohort	Australia	Not studied	Not studied	Not studied	Not studied	Not studied	Not studied	Not studied	Immune system activation	Not studied	Not studied	Not studied
Hornig M, et al. 2018	Cohort	Norway	Not studied	Not studied	Not studied	Not studied	Not studied	Not studied	Not studied	Sustained fever over time	Recurrent fever and in the second trimester	Not studied	Not studied
Mazina V, et al. 2015	Cohort	North America	Not studied	Not studied	Not studied	Not studied	Not studied	Not studied	Not studied	Infection and fever	Not studied	Prenatal history of infection and CNV	Not studied
Froehlich-Santino W, et al. 2014	Cohort	California	Not studied	Not studied	Not studied	Not studied	Not studied	Not studied	Not associated	Not studied	Respiratory distress, oxygen requirement, and unscheduled cesarean section	Not studied	Not studied

*Abbreviations:SSRIs* selective serotonin reuptake inhibitors, *GDM* Gestational diabetes mellitus, *ICP* Intrahepatic cholestasis of pregnancy, *HDP* Hypertensive disorders of pregnancy, *PE* Preeclampsia, *PP* Placenta previa, *PROM* Premature rupture of membranes, *HG* Hyperemesis gravidarum, *CNV* Copy number variation, *IL‑17A* Interleukin‑17A, *IL‑1β* Interleukin‑1β, *IL‑13* Interleukin‑13, *MCP‑1* Monocyte chemoattractant protein‑1, *GM‑CSF* Granulocyte–macrophage colony‑stimulating factor, *TNF‑α* Tumor necrosis factor‑α

### Psychosocial factors

#### Maternal educational level

The educational level of the parents was studied only in the mothers [14–16], comparing the years of schooling completed by the mother and the ASD manifested in their offspring. This factor shows a certain tendency in those mothers who had completed 13 or more years of formal education [14], as well as in the group presenting the lowest educational level [15]. Among the educational levels, it should be noted that both the technical and university educational levels represent the highest numbers, comprising 46.7% of the mothers in the population under study, compared to those with lower educational levels [14].

The educational level of women who developed preeclampsia and their risk of having children with ASD was also analyzed. The data show that women with a higher level of education who developed preeclampsia during pregnancy may have a greater association with having a child with ASD, compared to those who did not develop it, with an adjusted odds ratio (OR) of 2.36 and a 95% confidence interval (CI) of 1.18 [15].

#### Substance use during pregnancy

The use of various pharmacological agents during pregnancy and their relationship with diagnosed ASD have been analyzed in a broad population sample. The findings show that treatments prescribed for infections (antibiotics), bronchial asthma (albuterol, methacholine), diabetes mellitus (insulin and metformin used as prophylaxis), polycystic ovary syndrome (norethindrone used as prophylaxis), arterial hypertension (calcium channel blockers), infertility treatment (progesterone and cyclobenzaprine), and epilepsy (topiramate) could be associated with having offspring with ASD [17], even when used as prophylaxis in some cases. Vitamins and supplements, such as folic acid, have also been studied and have demonstrated a possible link with the condition [15, 18, 19].

Among the associated medications described, those prescribed for epilepsy have been well studied. One study in particular, reported an odds ratio (OR) of 1.60 for these medications, while for medications used in the treatment of mental health conditions such as selective serotonin reuptake inhibitors (SSRIs), an OR of 1.80 has been reported, also indicating that more than 50% of mothers who used these inhibitors as medications had a child with epilepsy. This association appears to be stronger when their use occurred during the first trimester of pregnancy [17]. Furthermore, the drug Levothyroxine, commonly used in treatments for hypothyroidism, was also studied, but it did not apparently show an association with ASD [17].

Some of the articles analyzed considered the consumption of harmful substances during pregnancy and its impact on ASD [16, 17, 20–22]. One of the substances studied was tobacco [16, 20, 21], consumed either passively or actively, finding consistent patterns between its consumption and the manifestation of ASD in infants. These studies indicate that exposure to tobacco smoke, either passive or active, is linked to a higher risk of ASD, manifesting particularly pronouncedly in children whose mothers were active smokers during pregnancy, compared to the non-smoker control group.

#### Stress and maternal mental health

In various studies, the maternal mental health status, determined either through instruments or by the diagnosis of pathologies such as depression, anxiety, or perceived stress, has been associated with the presence of chronic obstructive pulmonary disease (COPD) in offspring [14, 16, 17, 19, 21, 23, 24]. Furthermore, an investigation analyzed the maternal psychobiological condition, determined by measuring the cortisol level in plasma samples of 84 pregnant women at 15, 19, 25, 31 and 36 weeks of gestation. When this condition was related to the social communication questionnaire applied to the offspring at 5 years of age, a potential connection was found between this hormone and the cases of ASD present in boys, but not in girls [23]. It should be noted at this point that cortisol is a biomarker of chronic stress, so it can be used to objectively determine this condition.

Regarding maternal psychosocial disorders, a greater tendency for ASD has been observed associated with poor maternal mental health, demonstrated in studies that reported an OR of 1.80; 95% CI: 1.65–1.96 [17], as well as maternal psychiatric history [21] and psychosocial factors [14]. It is important to consider that, to date, no study has addressed mental health pathologies in a differentiated manner, which prevents the specific identification of disorders such as depression, anxiety, anguish, and panic attacks.

#### Socioeconomic level

There is little evidence in the articles reviewed that consider the socioeconomic level as a variable presenting a relationship with ASD. Only one article was found exploring a possible association in low social strata [21].

### Biological factors

#### Maternal age

In six of the articles included in the literature reviewed, maternal age was explored as a variable potentially linked to ASD [14, 16, 18, 20, 21, 24]. Some of those studies indicated that the maternal age range of 20 to 30 years was most frequently observed in analyses associated with ASD [14, 16, 21]. Likewise, one of these studies performed multivariate analyses and calculated adjusted odds ratios (ORs), reporting that the maternal age group between 26 and 30 years showed the highest proportion of diagnosed children; however, maternal age was not examined as a primary independent variable but was instead included as a covariate to adjust and interpret the other study variables [25].

#### Nutritional status during pregnancy

A total of nine articles that considered maternal nutritional status during pregnancy were analyzed [14, 16–19, 21, 24, 26–28]. It was found, in general, that women with high nutritional status and excessive weight gain during pregnancy showed consistent patterns linked to ASD in their offspring [16, 17, 19, 21, 24, 28]. Furthermore, serum leptin levels were measured in relation to maternal overweight and ASD, and it was found that this hormone increases in those cases where ASD symptoms are present in the offspring [26]. In two of the analyzed studies, no association was found with the variables described [18, 27].

#### Gestational age and birth weight

The analyzed articles included a gestational age of less than 35 weeks in newborns among the variables examined, reporting associations with the development of ASD [16, 17, 24, 25, 27, 29–32]. The cutoff of gestational age used varied in each of the reviewed articles. Some of them used ranges between 29 and 40 weeks [30], while others considered intervals between 22 and 31 weeks [31]. It is worth noticing that another study compared gestational age ranges, considering from week 24 to before week 37, in a large cohort covering a period between the years 1980 and 2009 [32]. In that study, the analyses reported differences in the frequency of ASD according to the gestational age ranges, describing variations in relation to term and post-term pregnancies [24, 32]. In addition, one article considered newborns with more than 42 weeks of gestation in its analysis, reporting statistically significant associations (*p* < 0.05) in this category [32].

When determining gestational age, a particularly notable incidence was observed among neonates classified as small for gestational age (SGA) and the development of ASD [24, 29], with the association increasing in SGA newborns with chromosomal disorders[24]. Regarding birth weight, several studies included this variable in their analyses [16, 17, 24, 25, 29]. Associations between ASD and low birth weight were reported if the newborn’s weight was less than 3,000 g (OR: 1.18). Likewise, in a study using disaggregated weight categories, an OR of 2.32 was described for neonates weighing less than 1,500 g and an OR of 3.43 for those weighing less than 1,000 grams [25].

#### Pathologies associated with pregnancy

Maternal pathologies examined in relation to ASD include metabolic and cardiovascular diseases. Several studies reported significant statistical associations (*p* < 0.05) between diabetes mellitus and ASD [14, 15, 17, 24, 27, 33], as well as with hypertension (*p* < 0.001) [24]. In one particular study, the reported OR for diabetes was 1.10, with no observed variations according to maternal treatment status [17].

Gestational pathologies such as preeclampsia, gestational diabetes, and pre-eclampsia occurring with premature rupture of membranes were analyzed in various studies as variables related to ASD [14, 15, 24, 27, 33]. Their results indicate that these pathologies may be associated with the diagnosis of the condition. Furthermore, one study examined pre-eclampsia with premature rupture of membranes in relation to motor and socio-emotional problems observed in offspring [27].

Acute pathologies such as fever and maternal infection were present in most mothers in the analyzed studies, and these variables were associated with children diagnosed with ASD (*p* < 0.05), with an OR of 1.28 [17, 19, 24, 34–36]. It is worth noting that in one study, fever was the variable with the strongest association, independent of the stage of pregnancy in which it occurred, both in the adjusted (*p* < 0.011) and in the unadjusted model (*p* < 0.045). Significant results (*p* < 0.005) were also reported during the second trimester of pregnancy. When fever occurred in three or more episodes during pregnancy, one study reported an OR of 2.15, and it was also noted that the use of acetaminophen could be a protective agent in treating fever, decreasing the probability of ASD [35].

#### Prepartum, intrapartum and postpartum complications

The studies also analyzed the connection between the delivery route and the development of ASD, finding some discrepancies in the associations, since two studies determined that delivery by cesarean section presented an association (*p* < 0.05) [24, 37], in contrast with two other investigations in which no such link was found [14, 27].

Furthermore, four studies explored the association of complications such as birth asphyxia [14, 17, 37] and prolonged labor, finding an interaction between these variables and the development of ASD (OR: 1.06, *p* < 0.05) [17].

Regarding the APGAR test evaluation, poor scores and neonatal asphyxia were studied, with both conditions showing a higher probability of presenting ASD in the offspring [14, 17, 19, 28, 37]. However, findings were heterogeneous across studies. Prematurity was also examined as a perinatal factor in multiple studies, with reported associations with ASD observed both independently and alongside other perinatal factors [15, 16, 24, 25, 29–32].

In relation to postpartum complications, one study showed that female newborns presenting with jaundice (*p* = 0.02) [17], may have an association with ASD, however, this differs from the results described in other studies [15, 37]. Among other conditions studied as possible agents linked to the development of ASD are respiratory distress (*p* = 0.04), hypoxia (*p* = 0.02), requirement of oxygen (*p* = 0.009) and neonatal intracranial hemorrhage (*p* = 0.167) [17, 24, 27, 37].

#### Possible biochemical markers

In the search for potential biochemical markers for ASD, serum leptin levels from umbilical cord blood have been studied, as well as the levels of various cytokines present in the maternal serum. One of the reviewed studies demonstrated that leptin levels are associated with the development of ASD (*p* = 0.002) [26].

Additionally, the cytokines IL-1β (OR = 2.31, 95% CI 1.16–4.60), IL-7 (OR = 2.28, 95% CI 1.20–4.33), IL-13 (OR = 2.42, 95% CI 1.29–4.55) and MCP-1 (OR = 2.09, 95% CI 1.03–4.24) were analyzed, which showed a higher probability of ASD with coexisting intellectual disability (ID), while in another study it was revealed that the cytokines GMCSF (OR = 2.06, 95% CI 1.03–4.11) and TNF-α (OR = 2.31, 95% CI 1.18–4.50) were associated with higher probabilities of ASD with ADHD [21]. Finally, another investigation also showed an association with the cytokine IL-17 A, which was elevated from the 20th gestational week onwards, in cases of ASD [18].

Another component studied was the relationship of the number of copies (CNVs) that contribute to the occurrence of ASD. The copies that have been studied so far are 16p11.2, 22q11.2, 1q21.1, 7q11.23 and 15q11-q13, and they have been shown to be strongly associated to ASD, especially in those cases where the mother was exposed to infections during pregnancy [36].

It is worth noting that one of the investigations innovatively studied ASD through neonatal dried blood by analyzing it through metabolomics, which could allow obtaining biochemical markers of ASD that may be present at birth, thus allowing a potential neonatal screening [22].

#### Biological sex of the fetus

A significant predominance of males presenting ASD was evident, a pattern that was consistently observed in a large number of studies [14, 16, 18, 19, 21, 23–25, 28]. One of the ways in which this predominance has been determined is through evaluation by means of ultrasound and the association with the diagnosis of ASC in the childhood period [19].

#### Factors not found in the articles analyzed

The literature shows that, to date, no studies have analyzed social factors such as violence, support during gestation and marital status as possible determinants for ASD.

## Discussion

This scoping review allowed us to map the available evidence on biopsychosocial factors associated with the diagnosis of ASD, identifying a wide variety of biological, perinatal, and psychosocial variables studied in different contexts. However, the methodological heterogeneity observed in terms of design, diagnostic criteria, measurement instruments, and variable control used in the analyzed studies is confusing, limiting comparability between them and, consequently, hindering the consistent interpretation of those findings.

In relation to psychosocial factors, the maternal educational level has frequently been reported as an associated variable. However, evidence suggests that this association may reflect broader structural determinants, such as unequal access to health services, timely diagnosis, and greater awareness of the importance of health checkups. Therefore, rather than a direct risk factor, the mother’s educational level may be acting as an indicator of social inequities in the detection and diagnosis of ASD.

Regarding SSRIs exposure during pregnancy, some studies reported associations with ASD; however, the results have proven inconsistent and methodologically diverse. In particular, there is little standardization in the characterization of the maternal psychiatric disorder, symptom control, treatment duration, and the presence of psychosocial comorbidities. Given that mental health disorders often coexist with chronic stress, socioeconomic vulnerability, and experiences of violence, it is difficult to isolate the potential pharmacological effect from the biopsychosocial context in which the exposure occurs.

Regarding maternal nutritional status, body mass index (BMI) has been reported as an associated variable in multiple studies. However, BMI is linked to metabolic, inflammatory, and cardiovascular alterations that could act as intermediary mechanisms. In this sense, current literature does not allow us to discern whether BMI constitutes an independent factor or a risk marker within a broader pathophysiological framework. Nevertheless, it is important to consider the implications this condition could have on the individual, due to the significant increase (*p* < 0.001) that BMI has shown over time, increasing by 0.34% (95% CI: 0.23–0.45) for each passing year [38].

Perinatal variables such as gestational age and birth weight were also frequently described. However, these outcomes often coexist with obstetric pathologies such as gestational diabetes or hypertensive disorders, which share inflammatory and angiogenic mechanisms. The overlap of these factors suggests the need for multivariate analytical models that allow for the understanding of complex interactions rather than isolated associations.

Finally, the diagnostic predominance in males was consistently reported. However, recent literature suggests that this difference could be influenced by diagnostic biases and a lower sensitivity of clinical instruments to detect the female phenotype, which could contribute to masking the condition in girls.

Taken together, this review demonstrates that the study of biopsychosocial factors associated with ASD is characterized by a predominantly fragmented approach. Therefore, there is a critical need for longitudinal research with robust designs, adequate control of variables, and integrative theoretical frameworks that consider the interaction between biological, psychological, and social determinants, along with a comprehensive characterization of the broad spectrum of ASD.

From a clinical and public health perspective, the findings reported in this review underscore the importance of adopting comprehensive approaches to prenatal and perinatal care, simultaneously considering biological, psychological, and social determinants. While the current evidence does not allow for establishing causality, the recurring identification of obstetric, metabolic, and psychosocial factors associated with early pregnancy loss, highlights the need to strengthen early screening strategies, timely prenatal care, and maternal mental health support.

These results also reinforce the importance of public policies aimed at reducing inequities in access to specialized care and promoting healthy prenatal environments, particularly in vulnerable populations. An interdisciplinary approach could contribute to earlier detection and more timely interventions in early childhood.

## Conclusions

The available evidence on biopsychosocial factors associated with the diagnosis of ASD is extensive but methodologically heterogeneous, comprising publications that describe its causes, addressed from genetic and epigenetic perspectives. However, this heterogeneity limits the possibility of establishing clear causal relationships. The reviewed studies suggest the involvement of multiple biological, perinatal, and environmental variables that interact within a complex and multifactorial context.

Among the most studied obstetric pathologies associated with ASC are preeclampsia, neonatal asphyxia, birth weight, and gestational age, all of which are factors that increase infant morbidity and mortality [39].

Tobacco use has been studied, revealing a possible interaction between exposure to tobacco smoke, whether passive or active, and ASC in children.

According to the reviewed literature and from an integrative perspective, the findings reinforce the need for longitudinal and multidisciplinary approaches that allow us to understand the interactions between biological, psychological, and social determinants.

It is crucial to understand the ASD as a condition of multifactorial origin. The growing relevance of fetal programming highlights the need for healthcare professionals to educate pregnant women about the importance of pregnancy planning and the conditions under which it takes place. It follows that pregnancy complications can help identify high-risk groups that could benefit from early detection and intervention, thus facilitating clinical follow-up.

### Limitations

This review identifies significant gaps in methodological standardization and variable control. Future research should prioritize robust designs that incorporate integrated biopsychosocial models to advance evidence-based prevention strategies and a better understanding of the ASD early development.

## Data Availability

All data analyzed in this study are included in this published article and its supplementary information files.

## Abbreviations

ASD: Autism Spectrum Disorder
ASC: Autism Spectrum Condition
SGA: Gestational age
ADHD: Attention-Deficit/Hyperactivity Disorder
